# Electrodialysis Using Zero-Gap Electrodes Producing Concentrated Product Without Significant Solution Resistance Losses

**DOI:** 10.3390/membranes15060186

**Published:** 2025-06-19

**Authors:** W. Henry Freer, Charles Perks, Charles Codner, Paul A. Kohl

**Affiliations:** Chemical and Biomolecular Engineering, Georgia Institute of Technology, Atlanta, GA 30332, USA

**Keywords:** electrodialysis, energy-efficient, nutrient recovery

## Abstract

Electrochemical separations use an ionic current to drive the flow of ions across an ion exchange membrane to produce dilute and concentrated streams. The economics of these systems is challenging because passing an ionic current through a dilute solution often requires a small cell gap to lower the ionic resistance and the use of a low current density to minimize the voltage drop across the dilute product stream. Lower salt concentration in the product stream improves the fraction of the salt recovered but increases the electricity cost due to high ohmic losses. The electricity cost is managed by lowering the current density which greatly increases the balance of the plant. The cell configuration demonstrated in this study eliminates the need to pass an ionic current through the diluted product stream. Ionic current passes only through the concentrated product stream, which allows use of high current density and smaller balance of the plant. The cell has three chambers with an anion and cation membrane separating the cathode and anode, respectively, from the concentrated product solution. The device uses zero-gap membrane electrode assemblies to improve the cell voltage and system performance. As ions concentrate in the center compartment, the solution resistance decreases, and the product is recovered with a lower voltage penalty compared to traditional electrodialysis. This lower voltage drop allows for faster feed flow rates and higher current density. Additionally, the larger cell gap for the product provides opportunities for systems with solids suspended in solution. It was found that the ion collection efficiency increased with current due to enhanced convective mass transfer in the feed streams.

## 1. Introduction

Population growth puts an ever-greater strain on resources creating the need for new methods of producing valuable products from wastewater streams. The recovery of nitrogen and phosphorous nutrients from waste streams has become increasingly popular. Nitrogen products especially are being targeted for recovery from waste streams for use as an agricultural fertilizer [1,2,3].

Many electrodialysis (ED) techniques have limited applicability due to their low current density [4,5,6,7]. ED techniques often have a limiting current density which limits the recovery rate from the system [5,8]. The low limiting current density drives up the electrode area (and balance of the plant) in order to meet commercial product demand. Previous studies have summarized ED as “not so economically competitive as other membrane separations” because of the high plant costs [9]. Most membrane electrode assemblies (MEAs) are not economical if they are operated at low current density [9]. Another issue with ED systems is the small cell gap required to keep the voltage drop across the dilute product stream manageable [4,5,6,7]. The cell gap in an ED system can range from 1 mm to 0.5 mm [3,5]. This small cell gap can limit the effectiveness of the cell because it is difficult for a large quantity of liquid to flow through a thin gap, especially if the stream contains suspended solids. Some wastewater inlets are not homogenous and can clog small openings easily [7]. The high density of solids in electrochemical waste activated sludge (EWAS) makes it especially important that the cell gap not be too thin [10]. The initial solution conductivity reported for EWAS feed solutions was between 1 mS/cm and 10 mS/cm, which decreased with time in an ED system as the salt products were extracted [4,6,7].

In this study, a new cell design is demonstrated which incorporates zero-gap electrodes and MEAs that avoid flowing ionic current through dilute solutions. Two zero-gap, porous electrodes were pressed against membranes forming the anode and cathode. A concentrated product stream was produced between the anode and cathode chambers because the membrane configuration forced ions to only flow into the center product chamber. Ionic current flows only through the highly conductive membranes and the concentrated product solution. By keeping the middle compartment concentrated, the voltage drop across the cell was minimized. Thus, high current density was achieved without having to use an extremely small cell gap for the product stream. The demonstration cell reported here used a 5 mm wide center chamber that could be adjusted without incurring an excessive voltage penalty.

## 2. Experimental

A Thermo Scientific Orion Lab Star EC112 (Waltham, MA, USA) Conductivity Bench Meter was used and connected to the Thermo Orion 011050MD 2-electrode conductivity cell. The conductivity probe was used to measure the conductivity of the feed solution and the final concentrated solution to determine the change in concentration based on conductivity. An Oakton Instruments Acorn pH 6 Meter was used to measure the pH of the feed solution before and after the tests. Power for the cell was provided by a Tek Power TP3005T (West Palm Beach, FL, USA) Regulated DC Variable Power Supply 0–30 V, 0–5 A. It was used under controlled voltage, where the voltage was set to a specified value. Dilute feed stock was an equimolar (0.1 M) solution of monopotassium phosphate (KH_2_PO_4_) and dipotassium phosphate (K_2_HPO_4_) (Sigma Aldrich) (St. Louis, MO, USA).

**Figure 1:**
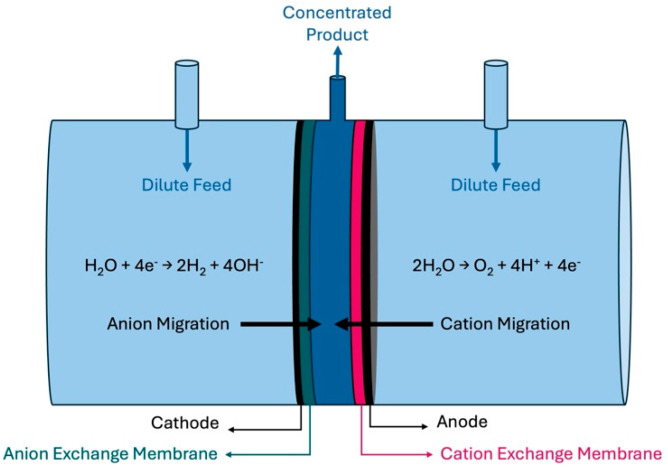
Concentrating cell diagram. Two outside chambers with dilute stock are separated by a middle chamber.

A three-compartment cell was used, as shown in Figure 1. The outside chambers, which were much larger than the inside chamber, received the dilute feedstock at a designated flow rate. The middle compartment was 0.5 cm in width and 5 cm in diameter. Current was applied to the cell producing hydrogen and hydroxide on the left side and producing oxygen and protons on the right side. The hydrogen-producing cathode was in contact with an anion exchange membrane (AEM) and the oxygen-producing anode was in contact with a proton exchange membrane (PEM). Charge balance between the two electrodes requires the net movement of negative ions to the right or positive ions to the left (or a combination of both). Since an AEM was used at the cathode, only negative ions will flow to the right from the outer feed compartment into the center product compartment. The PEM on the anode allows only positive ions to migrate, which causes a net flux to the left from the feed solution to the center product chamber. Thus, ions migrate into the center compartment (i.e., anions migrate from the cathode, and cations from the anode) to balance the charge in each compartment during electrolysis. The overall effect is for the product to accumulate in the center compartment during electrolysis. Water enters the center compartment due to an ion hydration shell from both sides: anions from the left and cations from the right side.

The feed stock was an equimolar (0.1 M each) solution of K_2_HPO_4_ and KH_2_PO_4_ because both molecules will dissociate in water to create a solution with available ions for migration into the center compartment while also acting as a buffer to maintain a relatively neutral pH. Using the Henderson–Hasselbach equation, Equation (1), the pH of this equimolar K_2_HPO_4_ and KH_2_PO_4_ solution was 7.2 [11].

(1) $$\mathrm{pH}={\mathrm{pK}}_{a}+\log\frac{\left[{\mathrm{HPO}}_{4}^{2-}\right]}{\left[H_{2}{\mathrm{PO}}_{4}^{-}\right]}$$

Calculating the expected flux of ions into the center of the cell involved converting the operating current to equivalents of electrons causing an equal number of equivalents of charge to migrate into the center compartment (Equation (2)). The flux of potassium and phosphate ions transported into the center compartment was calculated from the measured change in conductivity in the center compartment, as shown in Equation (3).

(2) $$\;\frac{I\cdot t}{F\cdot A}=\frac{{\mathrm{mol}\;e}^{-}}{{\mathrm{cm}}^{2}}$$

(3) $$\frac{∆\sigma \cdot x}{\Lambda_{\mathrm{total}}\cdot \gamma }=\frac{{\mathrm{mol}\;e}^{-}}{{\mathrm{cm}}^{2}}\left(\mathrm{Measured}\right)$$

In Equations (2) and (3), I is the current, t is time, F is Faraday’s constant, Δσ is the total change in conductivity, x is the cell gap, ∧ is the total equivalent conductance, and γ is the activity coefficient. The activity coefficient (γ) was obtained by dividing the measured conductivity by the theoretical conductivity (Table 1) [12].

**Figure 2:**
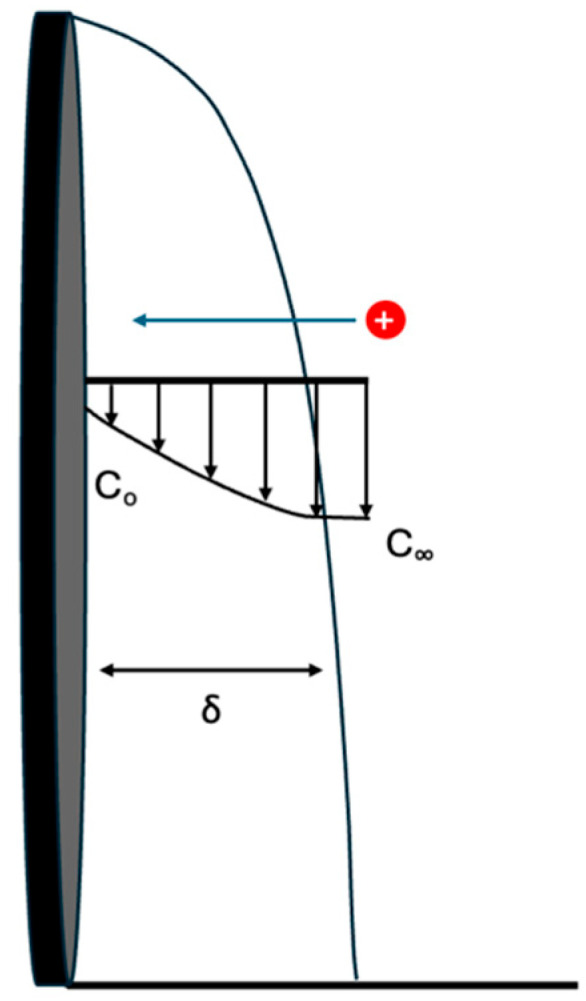
Boundary layer representation. The upper arrow shows direction of gradient and lower arrow shows width of depletion layer.

The zero-gap electrodes with a concentrated salt solution in the center chamber provided low ionic resistance because ionic current passed only through the concentrated product stream in the center compartment and not the feed solutions. However, the transport of ions from the feed solutions to the zero-gap electrodes occurred only by diffusion and convection, not migration. Figure 2 shows a diffusion concentration profile when the bulk solution is more concentrated than the surface of the electrodes. This diffusion profile has a flow across the surface and mass transfer perpendicular to the flow direction dependent on the boundary layer thickness (δ).

The boundary layer thickness was related to flow rate via the Reynolds number (Re), as shown in Equations (4) and (5). The boundary layer thickness has a lower velocity dependence in the turbulent regime, compared to the laminar flow regime. For this study, the water flow rate was moderate making the flow laminar. The flow rate used in this study ranged between 8 × 10^−8^ m/s and 4 × 10^−7^ m/s, which corresponds to a Reynolds number from 3 × 10^−4^ to 0.2. As velocity increased, the Reynolds number increased and the boundary layer thickness decreased. The relationship between mass flux and boundary layer thickness is given by Fick’s first law of diffusion shown in Equations (6) and (7).

(4) Re=ρvLμ

(5) δ ∝ 1Re

(6) $$J=k\left(C_{\infty }-C_{o}\right)$$

(7) $$k\propto \frac{D}{\delta }$$

In Equation (4), ρ is the density, µ is the viscosity, ν is the velocity, and L is the characteristic length for the flow system. In Equations (5)–(7), δ is the boundary layer thickness, J is molar flux, k is the mass transfer coefficient, C is concentration, and D is the diffusivity.

Osmosis is the transport of water from regions of low solute concentration to regions of high solute concentration across a membrane. As the middle compartment of the cell becomes more concentrated, water will move across the membranes from the more dilute outer chambers into the more concentrated center compartment by osmotic pressure. The equations for osmotic pressure are given in Equations (8) and (9). The reported literature value for the osmotic coefficient (φ) of KH_2_PO_4_ at a concentration of 1 M is 0.75 [13].

(8) $$\Pi =i_{\mathrm{eff}}\cdot C\cdot R\cdot T$$

(9) $$i_{\mathrm{eff}}=i\cdot \varphi$$

In Equations (8) and (9), C is the molar concentration difference of ions, R is the ideal gas constant, T is the temperature, and i_eff_ is the Van’t Hoff factor. The Van’t Hoff factor (i) can be adjusted by φ, which is the osmotic coefficient.

## 3. Results

**Figure 3:**
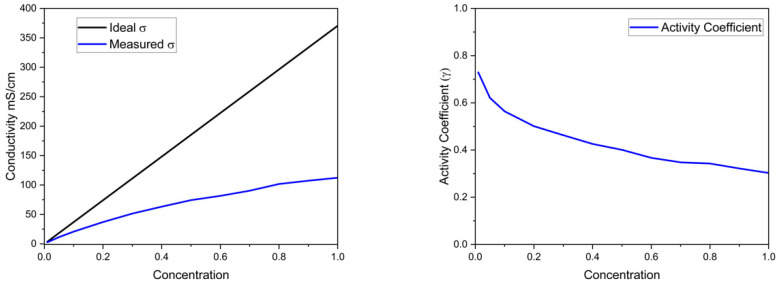
(**Left side**) Conductivity vs. concentration (lower line) with the dilute solution conductivity (upper line) based on molar ionic conductivity. (**Right side**) Activity coefficient as a function of concentration calculated by dividing the measured conductivity by the ideal conductivity.

Electrolysis was performed using a zero-gap anode (with a PEM) and cathode (with an AEM) to produce oxygen and hydrogen, respectively, and drive salt accumulation of product salt in the center compartment of the cell (Figure 1). The salt concentration in the center compartment was evaluated by measuring its conductivity and pH. A working curve for conductivity vs. concentration was created since the concentration deviates from activity at the concentrations used in the experiments. Figure 3 shows the actual conductivity vs. equimolar K_2_HPO_4_ and KH_2_PO_4_ concentration and the result for an ideal solution using the diluted solution values for equivalent conductance. The difference between the two is the resulting activity coefficient.

The measured conductivity for the equimolar KH_2_PO_4_/K_2_HPO_4_ solution deviated from the dilute solution equivalent conductance values, as expected. The difference between the measured conductivity and the dilute solution values is expressed as an activity coefficient, as shown on the right side of Figure 3. The activity coefficient vs. concentration was used as a working curve to obtain the concentration in the electrodialysis cell.

As described in the experimental section, the equimolar buffer solution was fed into the two outer chambers of the cell, and the center compartment was preloaded with the same starting solution. The conductivity of each compartment was measured as a function of electrolysis current and time to determine the salt concentration in each chamber of the cell. The conductivity of the initial solution was 24 mS/cm ± 0.5 for all experiments. The first experiment was to establish the reproductivity of the concentration measurement method and ensure that soaking in the membranes did not change the conductivity of the solutions. The cell was assembled with membranes, electrodes and salt solutions, and soaked for 3 hr. The conductivity of the each compartment was measured after 3 h, and all three chambers had values within the 0.5 mS/cm of the initial value. The pH of the cell remained the same in each compartment.

The next control experiment involved operating the cell without membranes at 2.5 V for 1.5 h. The average current during the experiment was 141 mA. The conductivity in the solution (i.e., one large chamber) was within the 0.5 mS/cm of the initial value and the pH was unchanged. The acid and base produced at the anode and cathode, respectively, were neutralized, resulting in no net change in the solution, and salt migration during electrolysis did not change the conductivity because the solution was well mixed.

**Figure 4:**
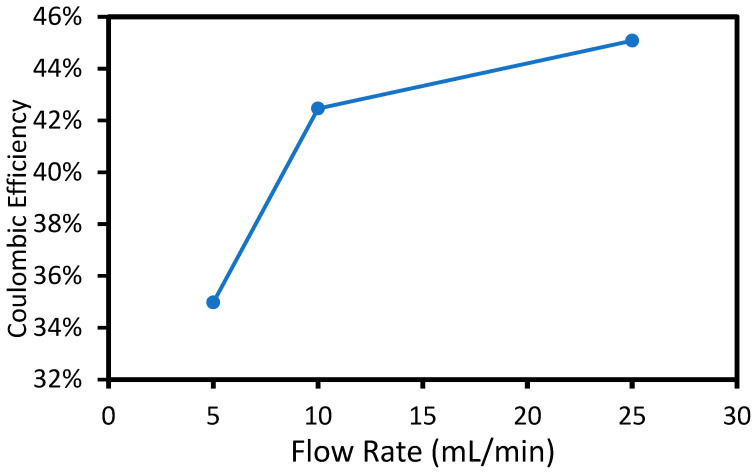
Flow rate vs. coulombic efficiency at 2.00 V cell potential. The average current was 17 mA and the uncertainty of the measured values was 2%.

The goal of this effort was to investigate whether it is possible to extract salts from dilute feed solutions (i.e., in the outer chambers) producing a concentrated product stream (i.e., in the center chamber) without suffering the enormous voltage penalty across the dilute product stream between the two electrodes which normally occurs in ED. The full cell was assembled with an equimolar KH_2_PO_4_/K_2_HPO_4_ solution in each compartment. The applied voltage was 2.00 V, and the flow rate of the feed solutions in the two outer chambers was varied. The concentration of the product stream (i.e., center compartment) was determined by measuring the conductivity and pH of the center product stream. The total charge passed was determined by integrating the current (average value of 17 mA) with time. The resulting coulombic efficiency (moles of salt accumulated in the center product chamber divided by the moles of electrons) is shown below in Figure 4.

The coulombic efficiency at 5 mL/min flow rate was 33%, which means that 33% of the charge resulted in migration and accumulation of KH_2_PO_4_/K_2_HPO_4_ salt product in the center chamber. The electric current caused ion migration in order to maintain charge neutrality. Only anions migrate through the AEM and only cations migrate through the PEM. The KH_2_PO_4_/K_2_HPO_4_ salt accumulation in the center compartment was lower than the expected, assuming only potassium and phosphate ions were mobile (i.e., 100% efficiency because H^+^ and OH^−^ ions also migrated into the center compartment. If H^+^ and OH^−^ migration occurred, it would not result in a conductivity change in the center compartment because they would react to form water. Thus, the remaining charge (i.e., 67%) was attributed to H^+^ and OH^−^ migration into the center chamber resulting in water formation and no net change in KH_2_PO_4_/K_2_HPO_4_ concentration. Ion migration occurred only between the two electrodes because there was no electric field within the outer chambers. Thus, the KH_2_PO_4_/K_2_HPO_4_ salts were transported to the MEAs in the outer chambers by diffusion and convection. The type of ions transported across the membranes during electrolysis depends on the concentration and mobility of each ion. An increase in flow rate in the outer chambers resulted in improved coulombic efficiency due to higher mass transport of KH_2_PO_4_/K_2_HPO_4_ to the anode and cathode by convection-enhanced diffusion, making them more competitive for migration into the center compartment. As discussed in the experimental section, the flow rate of the bulk solution in the outside chambers affects the boundary layer between the electrode surface and the bulk-stock solution in the outer chambers. The higher feed flow rate increased the average Reynolds number from 3 × 10^−4^ to 0.2 in the feed compartments, resulting in a decrease in the boundary layer thickness for the electrodes. By decreasing the boundary layer, the KH_2_PO_4_/K_2_HPO_4_ ions had a shorter distance to travel in order to diffuse to the membrane–electrode interface, which made them more competitive with H^+^ and OH^−^ migration into the middle chamber.

**Figure 5:**
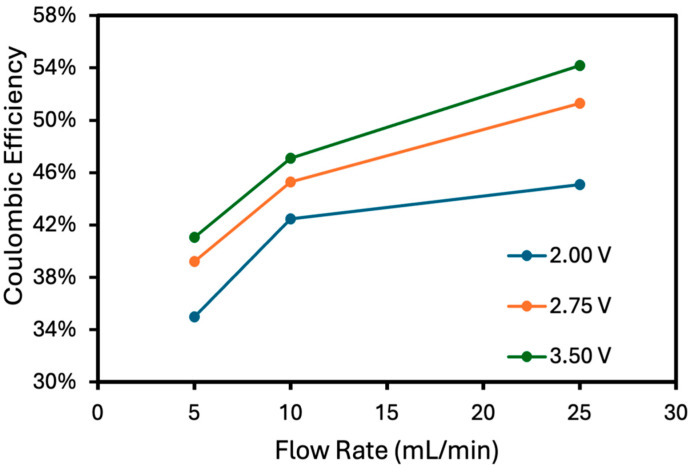
Flow rate vs. coulombic efficiency at 2.00 V, 2.75 V, and 3.50 V. The uncertainty of the measured values was 2%.

The three-flow-rate experiment (Figure 4) was repeated using different applied voltages, as shown in Figure 5. The average current at 2.75 V and 3.5 V is shown in Table 2. The current rose rapidly with applied voltage (non-linear) because the activation overpotential for water electrolysis decreases at higher overpotential. The added cell voltage was used primarily to overcome ionic resistance between the two electrodes.

The trend of higher coulombic efficiency with flow rate (constant voltage) shows the benefit of decreasing the diffusive boundary layer thickness. A thinner boundary layer improved the net flux (considering diffusion) to the two electrodes. The improved coulombic efficiency with flow rate in the outer chambers is expected because the diffusive boundary layer thickness (and thus mass transfer coefficient) scales with the square root of the velocity (flow rate). Any efficiency losses can be attributed to the competition for crossover with protons and hydroxide. Although the pH remained close to 7 due to the buffer, the local pH at the electrodes can deviate from that of the bulk solution. The protons that are made at the anode and hydroxides that are made at the cathode can cross the membranes and combine to form water in the center compartment, which does not result in product collection in the center compartment.

**Figure 6:**
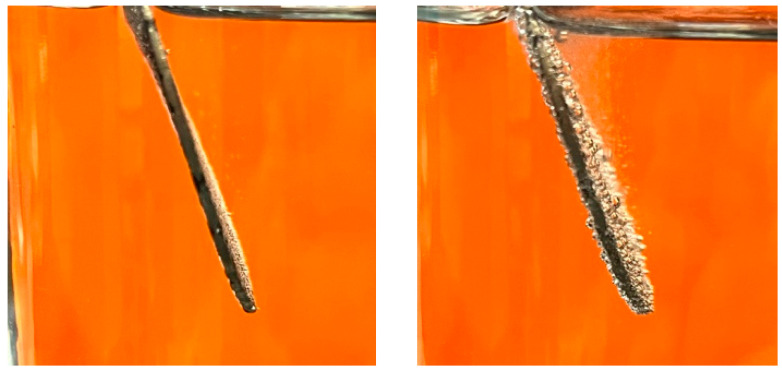
(**left**) Electrode surface at 2.00 V. (**right**) Electrode surface at 3.50 V.

In addition to improved coulombic efficiency with higher flow rate, there was also an increase in coulombic efficiency with current. Higher current resulted in higher overall ion flux into the center compartment. It is interesting that the higher overall flux also resulted in the KH_2_PO_4_/K_2_HPO_4_ salt being more competitive with H^+^ and OH^−^ migration into the center chamber (i.e., higher efficiency). The reactant feed into the anode and cathode chamber resulted in laminar flow across the surface of the electrodes. The higher current should increase the diffusive boundary layer thickness and decrease the diffusive mass transport coefficient. The data, however, shows the opposite effect on the coulombic efficiency. Instead, increasing the current (via higher voltage) improved the competitiveness of KH_2_PO_4_/K_2_HPO_4_ for migration into the product stream. This improvement in coulombic efficiency was due to contribution of convective mass transport from bubble formation during electrolysis. It was observed that at higher voltage, the bubbles formed from electrolysis of water were large and contributed significant convective mass transfer. This local agitation moved the mass transfer from the diffusion-dominated regime into the convective regime. As shown in Figure 6, the bubbles at the surface of the electrodes caused agitation at the surface of the electrodes, resulting in a decrease in boundary layer thickness and increasing the mass transfer coefficient.

KH_2_PO_4_/K_2_HPO_4_ electrolysis resulted in a higher salt concentration in the center compartment compared to the feed streams in the outer compartments. This raises the question of osmotic pressure-driven flow of water from the outer compartments into the center compartment. The osmotic pressure changes due to a concentration change from 0.1 M to 1.0 M in the center compartment is 16.7 bar, as seen from Equations (8) and (9). Thus, the osmotic pressure due to the concentration increase of the product stream caused water to permeate into the center compartment from the outer feed streams. However, this dilution effect of the product stream could be mitigated by pressurizing the middle compartment by the addition of a throttle valve on the product stream outlet. The burst pressure of the membranes used is typically in excess of 30 bar. The middle compartment could be operated at 30 bar to keep the osmotic flow below zero for a 1.0 M increase in concentration between the feed and product streams. In addition, a staged approach to the zero-gap electrolysis cell could be considered where the change in concentration difference between the feed and product streams is 1 M at each stage.

It is also interesting to consider the upper limit to the concentration of the product stream using this electrodialysis approach. Ultimately, the concentration limit of the product stream, without considering reverse osmosis dewatering of the product stream, is limited by the number of waters of hydration carried by each ion into the center product chamber. Potassium has six waters in its solvation shell [14]. HPO_4_^2−^ has a solvation shell of 12 at 1 M, and H_2_PO_4_^−^ has a solvation shell of 18 at 1 M [15]. Assuming an equimolar feed solution of KH_2_PO_4_ and K_2_HPO_4_, the maximum concentration of ions in the product chamber would be 6.7 M (8.25 waters per ion on average). If the product stream pressure was maintained at a value higher than the osmotic pressure (in a single- or multi-stage electrodialysis device), dewatering of the product could occur, resulting in concentrations higher than 6.7 M. The concentration increase as a final molarity and percent increase in concentration is shown in Table 3. The high voltage and fast flow rate concentrated the cell by 75%. Using Equations (8) and (9) and assuming an osmotic coefficient of 0.75, the osmotic pressure in the cell is 27.2 atm for KH_2_PO_4_ and 40.8 atm for K_2_HPO_4_ for a total osmotic pressure of 68 atm in the center compartment.

Hydrogen can be economically made by water electrolysis, as performed here, to drive the electrodialysis. The economic analysis of this system depends on the value of the concentrated salt product stream vs. the additional voltage required for ED (i.e., the voltage over and above that for electrolysis). To investigate the economics of this cell, the voltage drop across the cell at the three potentials used was measured and is reported in Table 4. Using the measured voltage drop across the cell due to solution and membrane resistance and the average current, the cost of salt separation was calculated. Attributing the full voltage drop across the membranes and electrolyte to ED is an overestimate because an electrolysis system would also have an ohmic voltage drop, although less than that encountered here. A price per kWh of $0.05 and cell efficiency of 54.18% (Table 2) was assumed, and the cost of salt production was found to be $0.97/kmol. Using the same cell gap and current density in a traditional ED configuration to produce 0.1 mM dilute water product would result in approximately a 1000× higher voltage penalty and electricity cost. This factor of 1000× electricity cost increase for traditional ED is unrealistic and would necessitate a change in cell design (a much thinner center compartment) and lower current density, driving up the size of the device and thus the cost of balance of plant.

A common salt that could be recovered is potassium nitrate. This cell’s price of $0.97/kmol is very competitive with the value of nitrogen used in fertilizer solution: $7.01/kmol N ($500/ton N) [16]. A more valuable salt product would be even more advantageous to collect. In the EWAS process, the recovery of fatty acids is also possible. A preliminary result using sodium acetate showed that the cell could concentrate carboxylate ions.

One of the clear advantages of this cell over traditional ED is that the cell can be used to concentrate ions between the electrodes, so the electrodialysis voltage penalty decreases as the cell operates and the product in the center compartment becomes more concentrated. ED often uses multiple feed and product solutions between the electrolysis electrodes so that one solution increases in concentration while the alternate one decreases in concentration. Compared to traditional ED, this zero-gap electrode has several advantages. First, traditional ED passes ionic current through dilute and concentrated product streams. One of the feed solutions between the electrolysis electrodes becomes more dilute with time, and the conductivity between the electrodes decreases. The voltage drop across the more concentrated product solution decreases; however, this is more than offset by the increase in the voltage drop across the dilute product stream. This increase in solution resistance scales with the concentration of ions, and recovering half the ions will thus double the resistance. After the first 50% of ions have been recovered, the same voltage penalty must be paid to collect only half as many ions as before, causing the solution resistance to quickly become an issue. The cell demonstrated here has a lower voltage drop across the product stream with time as the concentration of ions increased in the middle compartment, reducing the solution resistance. Second, as a result of the extreme voltage penalty across the dilute product stream, traditional ED is forced to use an extremely thin cell across the product stream, such as a cell with a width of 0.1 mm. This makes device configuration very challenging because it is hard to transition from large circular pipes to thin narrow slots, and small particles will easily clog the thin slots. In the zero-gap, concentrate-only cell demonstrated here, cell gaps in the order of cm can be used (100 times larger), making device construction easier and less sensitive to small particles. Third, the energy required per mole of product is proportional to the voltage drop across the ionic product solution. Traditional ED seeks to produce high-resistance, nearly pure water, whereas the zero-gap approach here is designed to produce a concentrated product whose conductivity is orders of magnitude higher. This allows for rapid removal of product at high current density. However, the zero-gap design has a lower coulombic efficiency (e.g., 50%), which raises the cost per mole of product. Last, the high current density and large cell gaps with the zero-gap design lowers the overall balance of the plant and the cost of maintenance, making it more scalable.

## 4. Conclusions

The zero-gap MEA three-chambered cell demonstrated here shows efficient salt separation into a concentrated product stream without the constraint of having to pass current through a dilute feed or product stream. Cell operation resulted in the concentration of ions in the center compartment, creating a more efficient cell over time. Once the middle compartment was concentrated to a satisfactory level, the cell could be run at steady-state conditions, where the salt product removed from the center would match the flow of salt and water into the center compartment. Operating the cell at higher current density and larger cell gap than most ED systems allows for a lower cost production of the product and the ability to handle higher flow rates without high membrane and electrode costs. Osmosis effects and local pH changes creating crossover competition are the main drawbacks of this configuration. Future studies will include running the cell at steady-state product concentration as well as investigating pH changes.

## Figures and Tables

**Table 1:** Molar ionic conductivity of ions in solution.

Ion	λ^0^ 10^–4^ m^2^ S/mol
K^+^	73.5
H_2_PO_4_^−^	36
1/2HPO_4_^2−^	57

**Table 2:** Coulombic efficiency vs. flow rate and voltage.

Dialysis Efficiency	Low Voltage (2.00 V)	Middle Voltage (2.75 V)	High Voltage (3.50 V)
Slow flow rate (5 mL/min)	34.97% 17 mA	39.20% 36 mA	41.04% 60 mA
Medium flow rate (10 mL/min)	42.46% 17 mA	45.30% 35 mA	47.10% 62 mA
Fast flow rate (25 mL/min)	45.08% 16 mA	51.28% 37 mA	54.18% 59 mA

**Table 3:** Final concentration and percent increase vs. flow rate and voltage. Initial condition 0.1 M KH_2_PO_4_/K_2_HPO_4_.

Dialysis Efficiency	Low Voltage (2.00 V)	Middle Voltage (2.75 V)	High Voltage (3.50 V)
Slow flow rate (5 mL/min)	14% 0.114 M	33% 0.133 M	57% 0.157 M
Medium flow rate (10 mL/min)	17% 0.117 M	37% 0.137 M	68% 0.168 M
Fast flow rate (25 mL/min)	17% 0.117 M	44% 0.144 M	75% 0.175 M

**Table 4:** Voltage drop across membranes at different voltages.

Applied Voltage (V)	Measured Voltage Drop (V)
2.00	0.075
2.75	0.231
3.50	0.295

## Data Availability

The raw data supporting the conclusions of this article will be made available by the authors on request.
